# Fhl5/Act, a CREM-binding transcriptional activator required for normal sperm maturation and morphology, is not essential for testicular gene expression

**DOI:** 10.1186/1477-7827-7-133

**Published:** 2009-11-24

**Authors:** Aurélie Lardenois, Frédéric Chalmel, Philippe Demougin, Noora Kotaja, Paolo Sassone-Corsi, Michael Primig

**Affiliations:** 1 Inserm, U625, Université Rennes 1, IFR140, Rennes, F-35042, France; 2 Biozentrum, Klingelbergstrasse 50-70, CH-4056 Basel, Switzerland; 3 University of Turku, Kiinamyllynkatu 10, FIN-20520 Turku, Finland; 4 University of California, Irvine, CA 92697, USA

## Abstract

**Background:**

The LIM domain protein Fhl5 was previously found to interact with CREM, a DNA binding transcriptional regulator necessary for spermiogenesis in mammals. Co-transfection experiments using heterologous promoter constructs indicated a role for Fhl5 in transcriptional up-regulation of CREM-dependent testicular genes. Male mice lacking Fhl5 were reported to be fertile but displayed partially abnormal sperm maturation and morphology.

**Methods:**

To identify Fhl5 testicular target genes we carried out two whole-genome expression profiling experiments using high-density oligonucleotide microarrays and total testis samples from Fhl5 wild-type versus homozygous mutant mice first in different and then in isogenic strain backgrounds.

**Results:**

Weak signal differences were detected in non-isogenic samples but no statistically significant expression changes were observed when isogenic Fhl5 mutant and wild-type samples were compared.

**Conclusion:**

The outcome of these experiments suggests that testicular expression profiling is extremely sensitive to the genetic background and that Fhl5 is not essential for testicular gene expression to a level detected by microarray-based measurements. This might be due to redundant function of the related and similarly expressed protein Fhl4.

## Background

Sexual reproduction of male mammals requires genes involved in meiosis, sperm formation and maturation as well as fertilization many of which are controlled by developmental stage-specific transcription factors [1,2]. Induction of post-meiotic gene expression is in part dependent upon the τ activator isoform of CREM (Cyclic AMP-Responsive Element Modulator) essential for spermatogenesis (for reviews, see [3-5]). Using a yeast two-hybrid assay CREM was shown to bind Fhl5 which is specifically expressed in meiotic spermatocytes and highly induced in post-meiotic round spermatids. Fhl5 is a protein containing four and a half LIM domains which are protein-protein interaction motifs found in many factors required for processes such as transcription, cell structure and motility as well as signal transduction [6-9]. The protein was shown to mediate strong reporter gene expression in transfection assays using heterologous promoter constructs in yeast and mammalian cells and its dynamic pattern of nuclear and cytoplasmic localization during early and late stages of spermiogenesis is mediated via direct interaction with the Kif17b kinesin motor protein [10-13]. Fhl5 is a member of a family of five related LIM domain proteins one of which, Fhl4, is also transcribed in testis showing peak induction in spermatids [11,14]. Fhl5 expression in pachytene spermatocytes and round spermatids was proposed to be dependent upon CREM not only in rodents but also in human; weak transcription of Fhl5 was found in three out of four infertile patients whose testes contained meiotic germ cells normally expressing the gene, suggesting a link between impaired Fhl5 function and spermatogenic arrest in a subgroup of individuals [15-18].

While CREM is essential for spermatogenesis, Fhl5 is not because mutant male mice were found to be fertile. However, they displayed partially abnormal sperm maturation and morphology which was suggested to be a consequence of impaired CREM/Fhl5-dependent post-meiotic gene expression [19]. Three testicular genes reported to be directly controlled by CREM continued to be expressed normally in the absence of Fhl5 but since numerous testicular transcripts were shown to depend upon CREM many candidate genes remained to be investigated [19,20].

To identify Fhl5 target genes, a whole-genome expression profiling experiment using total testicular samples and high-density oligonucleotide microarrays (GeneChips) containing probes for all known mouse protein-coding genes was carried out. We report that deletion of Fhl5 does not have a measurable effect on testicular gene expression at the level of sensitivity reached by microarray analysis when wild-type and Fhl5 mutant mice are examined in an isogenic background.

## Methods

### Mouse strains

The initial experiment was carried out with Fhl5-/- mice from a mixed genetic background (C57BL/6 and 129/SvPas) [19] as compared to wild-type mice described in reference [14]. The second experiment was done with Fhl5 +/+ and -/-littermates from a back-crossed strain (129/SvPas).

### PCR validation

Genotyping was done using allele specific primers as in reference [19].

### Testicular sample preparation

Decapsulated total testis samples were prepared from adult mice at the age of 9 weeks (C57BL/6 and 129/SvPas) as well as 9 and 14 weeks (129/SvPas) using a protocol as described [14].

### Target synthesis, GeneChip hybridization and raw data production

Total RNA preparation, cRNA target synthesis and raw data production using MG430 2.0 GeneChips (Affymetrix) was done as previously published [14,21]. Total RNA and cRNA quality was controlled using RNA Nano 6000 Chips and the BioAnalyzer; a virtual gel was created using a software option as recommended by the manufacturer (Agilent).

### Data processing and cluster analysis

Data analysis was essentially done as published using tools implemented in AMEN [14,22]. The data were normalized using the Robust Multi-array Average (RMA) method. Briefly, in the first experiment 23222 probe sets that yielded signals greater than 5.5 (median of normalized log2 transformed expression signals) were selected. Among 417 showing a fold-change across the samples ≥ 2 we identified 212 as significant with a LIMMA statistical test (F-value adjusted with False Discovery Rate ≤ 0.01). The genes were separated into two groups using k-means clustering.

In the second experiment 23921 probe sets were selected for which signal intensities >5.5 (median) were observed. Among those 21 showed a fold-change ≥ 2 but none was identified as reproducibly differentially expressed using LIMMA (F-value adjusted with FDR ≤ 0.01).

### MIAME compliance

Raw data CEL files are available for downloading via the EBI's ArrayExpress public repository [23] under the accession numbers E-TABM-130 (wild-type total testis control sample in the C57BL/6 and 129/SvPas background) [14] and E-TABM-806.

## Results

### The phenotype of Fhl5 -/- in a homogenous genetic background is exacerbated

It was previously reported that deletion of Flh5 in a mixed genetic background (C57BL/6 & 129/SvPas) did not impair fertility but resulted in reduced sperm counts, altered sperm motility and partially abnormal morphology [19]. To exclude the possibility that the complex background might mask a role of Flh5 in spermatogenesis a back-cross experiment was carried out and the testicular morphology of the resulting mice was investigated. The gene deletion phenotype in a homogenous background (129/SvPas) was more pronounced but the mice were still fertile (P. Sassone-Corsi, unpublished observation). We conclude that Flh5's role in sperm maturation and morphology is partially sensitive to the genetic background it is investigated in.

### Experimental procedure and quality control

**Figure 1:**
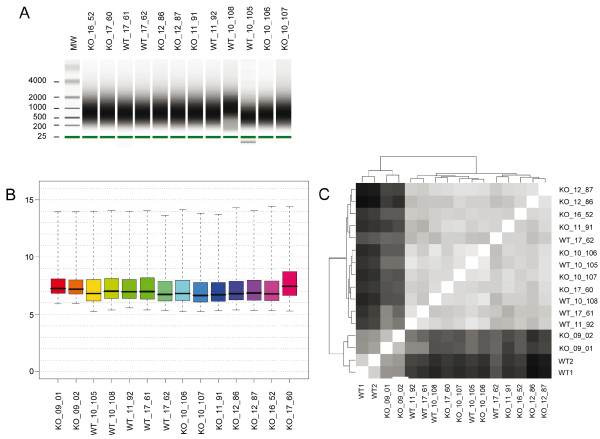
**Signal distribution and global similarity of samples**. Panel A gives RNA quality data shown as a virtual gel as indicated. A green bar is the internal standard. Molecular weight markers are shown (MW). The fragment size is given in bases. Panel B shows box plots of log 2-transformed signal intensities (y-axis) for the testicular samples as indicated (x-axis) before normalization (wild-type samples from the B6129SF2/J background not shown [14]). Panel C summarizes the overall degree of expression data similarity using a color-coded distance matrix (white is identical, back is most dissimilar). A dendrogram (top and left) shows the pattern of sample clustering observed.

To identify Flh5 target genes we first obtained decapsulated total testicular samples from mutant mice (C57BL/6 & 129/SvPas) whose genotype was verified by PCR and Western blotting (not shown). For practical purposes and cost-effectiveness cRNA targets were synthesized from the mutant mice testes, hybridized to MG430 2.0 GeneChips and compared to testicular and cell-type specific data from B6129SF2/J wild-type mice already available in our laboratory [14]. We detected weak differential expression for a very small number of genes that appeared however completely unrelated to post-meiotic male germline functions and known CREM targets. Therefore we next sought to exclude the possibility that differences in RNA concentration were due to heterogeneous genetic backgrounds between wild-type and mutant samples rather than the deletion of Fhl5. The experiment was repeated using Fhl5+/+ and -/- littermates from a homogeneous back-crossed strain (129/SvPas). The total RNA and cRNA samples were homogenous and of good quality (Figure 1, panel A) and the signal intensity distributions were in the normal range across the dataset (panel B). Overall signal similarity was studied using a distance matrix in combination with a dendrogram. The samples from the mixed background were set off but all other samples appeared to be very similar overall and no clear separation of wild-type versus mutant testis was detected (panel C).

### Flh5 is not essential for testicular gene expression in adult mice

**Figure 2:**
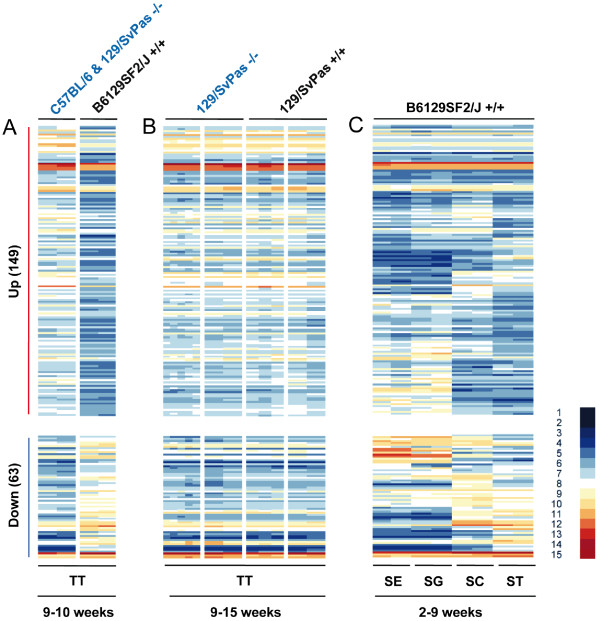
**Expression profiling of FHL5 mutant (-/-) versus wild-type (+/+) testes**. Panel A shows a color-coded heatmap for two groups of genes (up and down-regulated) as indicated by red and blue vertical bars when C57BL/6 & 129/SvPas -/- and B6129SF2/J +/+ were compared. Panel B displays data for these groups obtained in a different sample set as shown. Panel C displays the data obtained with purified testicular cells. The strain background is given at the top for wild-type (+/+) and mutant (-/-) samples. The age distribution of the mice is given. TT is total testis. Samples are in panel A column 1 (KO_09_01, KO_09_02), column 2 (WT_1 and WT_2), panel B column 1 (KO_10_106, KO_10_107, KO_11_91, KO_12_86, KO_12_87), column 2 (KO_16_52, KO_17_60), column 3 (WT_10_105, WT_10_108, WT_11_92) and column 4 (WT_17_61, WT_17_62). Panel C shows duplicate samples of Sertoli cells (SE), spermatogonia (SG), spermatocytes (SC), and spermatids (ST) purified from animals at the appropriate ages [14]. The color code for log2-transformed signals is indicated by a scale to the right of the heatmap ranging from the weakest signal 1 (dark blue) to the strongest signal 15 (dark red).

In the first experiment we compared two testicular samples from non-isogenic Fhl5 mutant and wild-type mice at the age of 9-10 weeks and identified among 45101 probe sets a set of 23222 cases displaying signals greater than 5.5, the median of the normalized log2 intensity values which we considered the cut-off for reliably detectable expression. We then employed the statistical LIMMA test to select 212 probe sets showing a fold-change greater than 2 (F-value adjusted with FDR ≤ 0.01). For further interpretation these probe sets were grouped into two clusters using a k-means algorithm that distinguished transcripts for which signals were either stronger (149) or weaker (63) in the mutant as compared to the wild-type strain. No Gene Ontology terms [24] were found to be significantly enriched among the two expression clusters and most genes affected were not expressed in spermatids where FHL5 exerts its function as an activator (Figure 2, panel A and C). To examine if these differences were due to distinct genetic backgrounds of wild-type and mutant mice rather than deletion of FHL5 *per se*, total RNA was prepared from back-crossed mice's total testis samples at 9-15 weeks of age. Strikingly, no differentially expressed genes were detected and as a consequence none of the genes first identified as up- or down-regulated showed any change of transcription between wild-type and mutant samples from animals that were litter mates (Figure 2, panel A and B).

**Figure 3:**
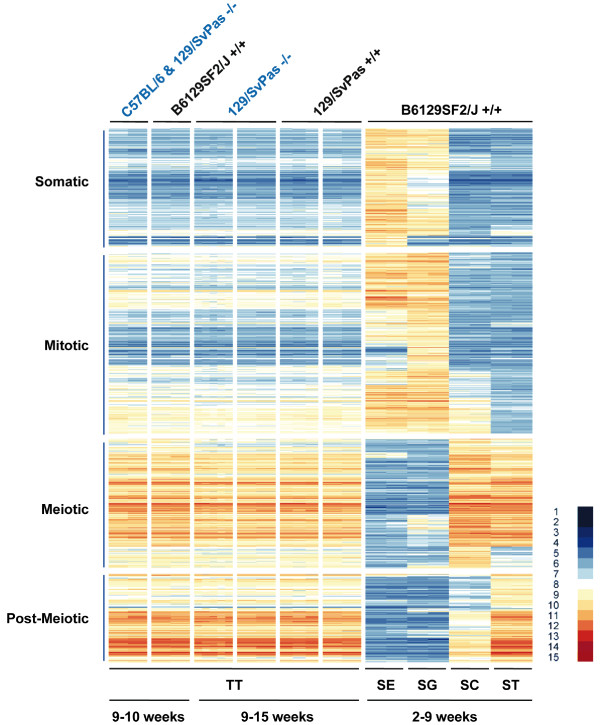
**The pattern of genes Differentially Expressed in Testis (DET) in FHL5 wild-type versus mutant samples**. The DET group was identified in [14]. A heat map is shown that displays the expression signals in testis in the presence and absence of FHL5 for genes showing peak expression in Sertoli cells (Somatic), spermatogonia (Mitotic), spermatocytes (Meiotic) and spermatids (Post-Meiotic). Samples and scale are as in Figure 2.

**Figure 4:**
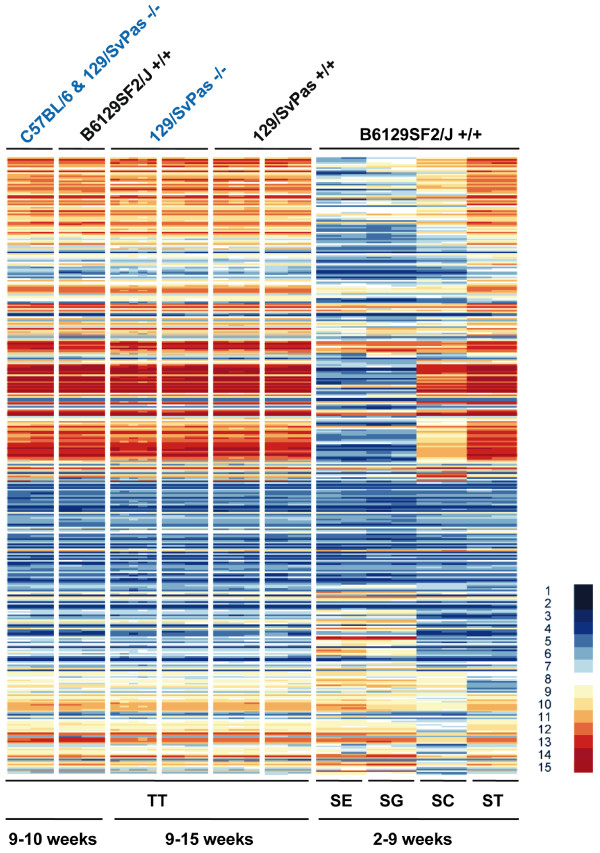
**The pattern of CREM-dependent genes in FHL5 wild-type versus mutant samples**. A heat map summarizes expression signals in testis in the presence and absence of FHL5 for genes reported to be transcriptionally dependent upon CREM [20]. Samples and scale are as in Figure 2.

We next investigated the expression patterns of genes previously identified as being differentially expressed in testis (DET) using enriched Sertoli cells, spermatogonia, spermatocytes and spermatids [14]. None of the genes falling into four somatic, mitotic, meiotic, and post-meiotic expression clusters (notably the post-meiotic one) displayed altered expression in the absence of FHL5 (Figure 3). Finally, we analysed the expression of genes found to depend upon CREM within our dataset and again detected no difference between wild-type and mutant samples (Figure 4) [20].

## Discussion

Fhl5 has initially been identified as a CREM-binding factor required for gene activation in the male germline. It was our aim to verify the hypothesis that abnormal sperm maturation and morphology observed in mutant mice lacking Fhl5 was due to impaired CREM-dependent gene expression. To this end we carried out two independent profiling experiments using total testicular samples and MG430 2.0 GeneChips covering all known protein-coding genes of the mouse genome.

The first experiment was based on total testicular samples from adult nine week-old Fhl5-/- mice produced in a mixed genetic background (C57BL/6 and 129/SvPas) [19] that were compared to data previously obtained with wild-type samples isolated at the same age but from another strain (B6129SF2/J) [14]. We considered this cost-effective and time-saving approach acceptable in spite of the influence of genetic backgrounds on gene expression which is especially pertinent in the case of human samples [25] because Fhl5 was a potent activator in transfection experiments [10,26]. However, among the genes we found in our initial experiment using Fhl5+/+ and -/- samples from different backgrounds none played a role in spermiogenesis, sperm maturation or fertility and not a single one fell into the group of CREM target genes previously identified by microarray analysis [20]. A detailed second expression analysis of litter mates failed to yield any differentially expressed genes in 12 samples from adult mice including five wild-type and seven mutant animals. It should be emphasized that these mice stemmed from a back-crossed strain background that displayed a stronger phenotype than the Fhl5 deletion in the initial mixed background (P. Sassone-Corsi, unpublished observation).

The most obvious explanation of these results is that the loci identified in the first experiment showed distinct expression patterns because we compared samples from different genetic backgrounds while the second experiment based on isogenic litter mates revealed that Fhl5 is not essential for testicular gene expression at the level of sensitivity achieved with microarrays and whole gonad samples. This result is in keeping with a previous report that the absence of Fhl5 has no effect on the expression of known CREM targets such as TNP1, PRN1 or PRN2 as assayed by PCR [19]. It also concurs with the fact that point mutations in the human ortholog of Fhl5 occur with the same frequency in fertile individuals and azoo- or oligozoospermic patients which argues against an essential role for the protein in human spermatogenesis [18,27]. It is possible that Fhl5's *in vivo *role as a transcriptional regulator is too subtle to be detected by whole-genome profiling using total testicular samples and that highly enriched populations of round spermatids would have to be used instead. We consider this unlikely, however, since Fhl5 was reported to be a strong activator and approximately 60% of the adult testicular cell mass consists of spermatids [10].

An alternative explanation is that a related factor to a large degree compensates the deletion of Fhl5 such that transcriptional effects are masked while sperm maturation and morphology remain partially abnormal. One likely candidate is the LIM-domain protein Fhl4 which was initially shown to be expressed in testis [11,28] and later found to be absent in spermatogonia and highly induced in spermatocytes and spermatids by microarray analysis [14]. Fhl4 weakly activates a heterologous reporter construct in yeast and mammalian cells but it cannot, as opposed to Fhl5, bind to CREM in a yeast two-hybrid assay [11]. However, this does not exclude the native Fhl4 protein from being able to partially take over roles of Fhl5 in the physiological context of adult testicular tissue, especially in the absence of functional Fhl5.

## Conclusion

The corollary of our work is that deletion of Fhl5 does not cause any significant changes in testicular expression at the genome-wide level at a level detectable by high-density oligonucleotide microarray expression profiling when compared to an isogenic wild-type sample. We speculate that this is the case because Fhl5 is redundant with Fhl4, a structurally related factor showing a similar testicular expression pattern. If this idea were correct, mice lacking Fhl4 should display a phenotype reminiscent of the one reported for Fhl5 mutants while deletion of both genes should disrupt spermatogenesis at the post-meiotic stage because of impaired CREM-dependent gene expression.

## Competing interests

The authors declare that they have no competing interests.

## Authors' contributions

AL processed and interpreted the data and contributed to the manuscript. FC contributed to data analysis. PD processed the samples and produced the raw microarray data. PSC and MP designed research and MP interpreted the data and wrote the paper. All authors have read and approved the final manuscript.
